# Letter from the Editor in Chief

**DOI:** 10.19102/icrm.2019.101004

**Published:** 2019-10-15

**Authors:** Moussa Mansour


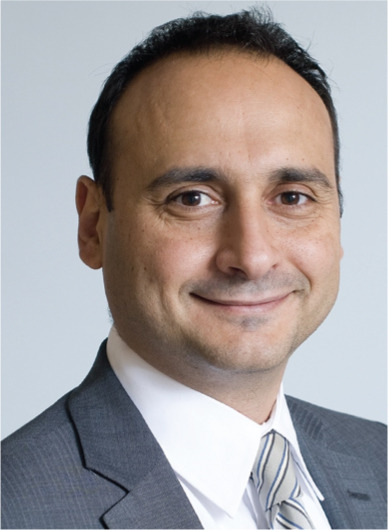


Dear Readers,

The field of left atrial appendage closure (LAAC) for atrial fibrillation (AF) continues to expand. Recently, the findings of a landmark study, the LAAC vs. Novel Anticoagulation Agents in AF (PRAGUE-17) trial (ClinicalTrials.gov identifier no. NCT02426944), were presented by Dr. Vivek Reddy at the Transcatheter Cardiovascular Therapeutics scientific meeting in San Francisco on September 26, 2019.

PRAGUE-17 is a multicenter, prospective, randomized, noninferiority trial conducted in 10 sites in the Czech Republic that compared LAAC and novel oral anticoagulation (NOAC) in high-risk AF patients. The patient population consisted of subjects with nonvalvular AF in addition to one of the following: (1) history of bleeding requiring intervention or hospitalization, (2) history of a cardioembolic event while taking anticoagulation, or (3) CHA_2_DS_2_-VASc score of three points or more and HAS-BLED score of two points or more. The postprocedure antithrombotic treatment consisted of dual antiplatelet agents for three months, followed by aspirin. The primary endpoint of the study was a composite of stroke or transient ischemic attack (TIA), systemic embolism, clinically significant bleeding, cardiovascular death, and significant periprocedural or device-related complications. The primary hypothesis of the study was that LAAC is noninferior to NOAC for the primary endpoint.

A total of 402 patients were enrolled in the study, evenly split as 201 patients in each arm. LAAC was performed using the Amplatzer™ Amulet™ (Abbott Laboratories, Chicago, IL, USA) or the Watchman™ device (Boston Scientific, Natick, MA, USA), and implantation was successful in 97% of the study participants who underwent said procedure. In the NOAC group, the majority of included patients were treated with the standard dose of apixaban. Procedural complications occurred in nine patients (4.8%) in the LAAC group and included two deaths. The main finding of the study was that the primary endpoint was met, demonstrating that LAAC is noninferior to NOAC for a composite outcome of cardiovascular death, stroke/TIA, systemic embolism, clinically significant bleeding, and significant procedure/device-related complications.

The investigators of PRAGUE-17 should be commended on the design and the completion of this pioneering study, which will likely have a significant impact on the field of stroke prevention in AF. The most immediate effect will perhaps be the catalyzation of the efforts to start a larger multicenter study comparing LAAC and NOAC in the United States, which we eagerly await.

Sincerely,


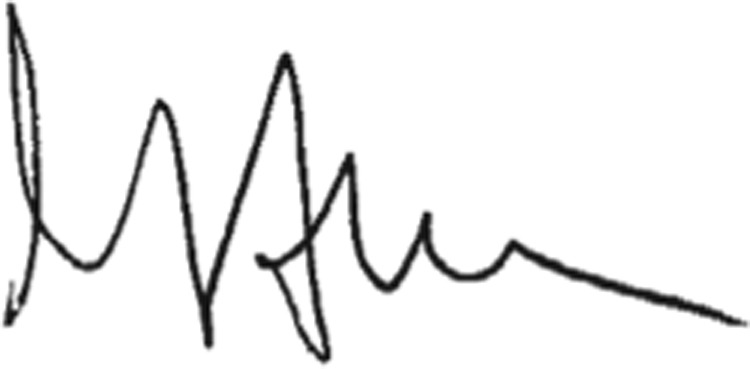


Moussa Mansour, md, fhrs, facc

Editor in Chief

The Journal of Innovations in Cardiac Rhythm Management

MMansour@InnovationsInCRM.com

Director, Atrial Fibrillation Program

Jeremy Ruskin and Dan Starks Endowed Chair in Cardiology

Massachusetts General Hospital

Boston, MA 02114

